# Mental Health and Coping in Women With ADHD During the Pandemic

**DOI:** 10.1177/10870547261437893

**Published:** 2026-04-14

**Authors:** Rebecca Nurgitz, Carlin J. Miller

**Affiliations:** 1 Hamilton Health Sciences, ON, Canada; 2 University of Windsor, ON, Canada

**Keywords:** ADHD, women, mental health, coping, pandemic, emotional insight

## Abstract

Recent research suggests that women make up a substantial proportion of adults with ADHD, but they are less likely to be identified, diagnosed, and adequately treated.

**Objective:**

A diverse group of 307 females (17–30 years of age) was recruited for an online mixed methods study on mental health and coping in women with ADHD.

**Methods:**

In the first phase of the study, data from women reporting low and high levels of ADHD symptoms, including some previously diagnosed with ADHD, were collected. Quantitative results suggest that women with substantial ADHD symptoms experience more difficulty with emotional regulation, more distress, greater loneliness, and they are less resilient and less self-compassionate. To follow up, all women who reported a previous diagnosis of ADHD in the first phase were invited to participate in interviews exploring how they cope with high stress. A total of 15 women were interviewed.

**Results:**

Qualitative results suggest that although participants described engaging with and regulating internal experiences as more effective, they were more likely to use avoidance coping, often thought to be less effective. Further, when participants reported less emotional insight/awareness, they were also less likely to use internally focussed approach strategies.

**Conclusion:**

Thus, the reduced self-awareness that is characteristic of ADHD may represent an additional challenge to effective coping. These results underline the importance of ongoing research and clinical attention for women with ADHD.

## Introduction

Originally conceptualised as a childhood psychiatric disorder affecting mostly boys, ADHD is now understood as a neurodevelopmental disorder that may continue to cause significant impairment into adulthood in both sexes. Characteristic symptoms of this heritable and prevalent disorder in later adolescence and adulthood include difficulty paying attention, poor impulse control, and a sense of restlessness (American Psychiatric Association, 2022). Research suggests that women are far more likely to have difficulties associated with distractibility and mind wandering (Attoe & Climie, 2023). These symptoms may cause impairment or distress in a variety of environments, including educational settings, workplaces, social interactions, and romantic relationships with a range of outcomes (Merrill et al., 2020; Rosello et al., 2020).

More recently, it has become clear that women and girls may be at greater risk for ADHD than previously thought. Indeed, recent studies suggest that females with ADHD are less likely to be diagnosed than males at all developmental stages (Hinshaw et al., 2022). This may be because ADHD has long been assumed to principally impact males, and the emphasis has often been on externally observed symptoms such as hyperactivity which are less common in females (Attoe & Climie, 2023). It may also be that symptoms in some females are more likely to be predominantly attention-related, which are less disruptive and thus, less noticeable at home, in classrooms, and in the workplace (De Rossi et al., 2022; Meyer et al., 2020) or symptoms may be misattributed to other disorders such as anxiety disorders (Coxe et al., 2021). Yet, there is also clear evidence to suggest that because of the stigmatisation of externalising symptoms in females, girls, and women with ADHD may face particular challenges (Visser et al., 2024). Thus, it is critical that research attention be focussed on the experiences of females with ADHD.

Behaviours associated with ADHD impact social interactions. For example, individuals with impulsivity and hyperactivity may be perceived as intrusive and overbearing by others, whereas fluctuations in attention may limit one’s ability to notice social cues and attend to the emotional states of others (Ragnarsdottir et al., 2018). Other contributors to social functioning difficulties in ADHD include difficulties with turn-taking, reduced social information processing, and deficits in emotion recognition (Ros & Graziano, 2018). Social difficulties in adults with ADHD in various domains are often attributed to emotional lability (Corbisiero et al., 2017). Furthermore, difficulty controlling intense emotional reactions and subsequent behaviours can be disruptive, overwhelming, and create tension in interpersonal relationships (Rosello et al., 2020). Thus, the problematic behaviours that characterise ADHD in many adults, such as emotional dysregulation, inattention, and restlessness hinder the ability to create and sustain meaningful relationships with others, which increases feelings of loneliness (Jong et al., 2024). These difficulties likely contribute to harsh self-criticism, which then negatively impacts one’s ability to cope during times of adversity (Ewert et al., 2021). When these difficulties intersect with cultural expectations for females to be socially skilled (Tan et al., 2018), women may be at particular risk for poor outcomes.

### Models for Coping

There is a robust base of theoretical models for coping that have been tested in empirical studies. Early models focussed more on dichotomous systems and typologies, such as the problem-focussed coping vs. emotion-focussed coping model (Lazarus & Folkman, 1984). Following from the simple models of coping, early measurement focussed on neat quantification of how coping was measured. More recent models have allowed for greater complexity, such as direction of movement relative to the problem (i.e., approach-avoidant), perceptibility of responses (i.e., overt-covert), and the impact of actions (i.e., direct-indirect; Chun et al., 2006). These more complex models also require greater complexity in measurement.

Adding to the complexity is the consideration of the intersection of coping with emotion regulation. Some researchers argue that coping is synonymous with emotion regulation (Suchy et al., 2019), while others consider them distinct constructs (Gross, 2014). Resilience, emotion regulation, and coping are distinct terms that converge both practically and theoretically. For the purposes of the current study and based on the review of the recent literature, coping refers to the specific strategies used to negotiate and manage difficulties. Comparatively, resilience refers to the capacity to respond adaptively to hardship to achieve the desired outcome (Liu et al., 2020). In contrast, emotion regulation involves the acute management of affective states regardless of the valence (i.e., positive or negative; Gross, 2014). In other words, coping is an umbrella term that includes emotion regulation as a strategy. Resilience is effectively the outcome of successful coping—the ability to respond flexibly to challenges and recover from hardship (Liu et al., 2020). Notably, there has been some debate in the literature about whether resiliency is appropriate to consider when there is not clear evidence for trauma or substantial adversity (Luthar & Cicchetti, 2000), an issue that is likely beyond the scope of this study. In summary, these constructs are complex and dynamic phenomena that are not easily captured using quantitative method.

Because the construct of coping is likely multidimensional and theorists have not agreed on a single conceptual model, extant quantitative measures of coping likely fail to capture its complexity (Kapsou et al., 2010). Thus, a qualitative approach may be better suited when evaluating the intersection of ADHD symptoms and coping during a highly stressful time, such as the recent pandemic.

### COVID-19 as a Context for Coping

COVID-19 was initially described as a global pandemic in March 2020, which led to extreme efforts to reduce community transmission, including the months-long closing of schools and businesses, physical distancing measures, and orders to stay at home and/or quarantine—all of which resulted in widespread decline in mental health and coping. A spate of research has demonstrated the adverse effects of pandemic-related isolation and loneliness (e.g., Dragioti et al., 2022). For instance, extended periods of isolation were found to be associated with greater psychological symptoms, including stress, anxiety, insomnia, depression, and suicidality (Lu et al., 2022). Although the restrictions associated with life during the pandemic have ended, many people report ongoing issues with distress that began during that time (Lu et al., 2022) and public health officials have warned that future pandemics are likely as the climate changes and the world’s population expands into territories previously inhabited by wildlife (Casadevall, 2024).

Loneliness, defined as a distressing experience due to dissatisfaction with the perceived quality of one’s interpersonal relationships (Betts & Bicknell, 2011), was a particular concern during the pandemic given the limitations placed on social gatherings and in-person interactions. The recommended and/or mandated models for social contact were limited to remote and distanced gatherings—both of which denied the opportunity for physical expressions of connection, including handshakes and hugs. In other words, although people were still able to interact with others in some ways, the available means of doing so was not necessarily sufficient to quash feelings of loneliness (Killgore et al., 2020). Further, people with ADHD may have been more vulnerable to the adverse effects of pandemic loneliness, as they may have been at higher risk for difficulties such as distress intolerance, emotional lability, and interpersonal problems (Bailie & Linden, 2024), as well as greater baseline loneliness (Houghton et al., 2020).

Prior to the pandemic, there were substantial direct and indirect costs of ADHD on healthcare systems, individuals, families, and communities (Chhibber et al., 2021). During the pandemic, people with ADHD sought healthcare more often than people without ADHD (Butt et al., 2023), perhaps overburdening a system already in crisis. There is substantial evidence to suggest a beneficial impact of treating ADHD in terms of reducing overall impairment and accidental injuries, as well as reducing risk for communicable infections like COVID (Merzon et al., 2021).

### The Present Study

The present study sought to describe the mental health of women with ADHD, including their coping strategies, emotional regulation, and loneliness, in the context of the highly stressful pandemic (June-November 2021). The study used a mixed methods design with initial quantitative data analyses comparing women with and without substantial ADHD-related symptoms on the variables of interest. In the second phase of the project, women with previous diagnoses of ADHD in the initial sample were invited to participate in qualitative interviews about their experiences with distress, loneliness, and emotion regulation during the pandemic.

Rather than predetermining sample size in qualitative research, the emphasis has been on achieving theoretical or thematic saturation—the point at which no new codes or themes emerge (Sandelowski, 1995). Although some have suggested a finite number (such as *N* = 12 in Fugard & Potts, 2015), others have noted that determining an a priori sample size is problematic because the main themes are not known in advance (Sim et al., 2018). As a result, all eligible participants from the first phase of the project were invited to participate in the second phase of the project.

## Methods

### Participants

Initially, 392 female participants were recruited for this study. As described below, they were assigned to two subgroups: those at low risk for ADHD and those at greater risk for ADHD. The resulting sample for the quantitative stage of the study included 307 females between the ages of 17 to 30 years old with a mean age of 21.82 (*SD* = 3.23). Respondents were living in Canada at the time of their participation and were White or of European descent (58.5%). In total, 237 participants were recruited from the university research pool and 70 participants were recruited from the community. Reflecting the predominance of undergraduate students in the sample, most were either unemployed (27.0%) or employed part-time (57.3%). All had completed their secondary education and 34.0% were working towards a post-secondary or advanced degree. Most were living with family members or a romantic partner at the time of their participation (81.1%).

For the qualitative phase of the project, participants were eligible if they reported having a previous ADHD diagnosis in the quantitative phase of the project, were between the ages of 17 and 30 years, able to read and understand English, and were Canadian or living in Canada at the time of the study. Eligible participants were recruited from both university and community samples. Of the 58 respondents who met the inclusion criteria and agreed to be contacted for future research, 15 female participants completed the follow-up survey. Participants in the second phase were between 19 and 30 years old with an average age of 24 years (*SD* = 3.28). Respondents identified as Non-Hispanic White or of European descent (66.7%, *n* = 10), Aboriginal/Indigenous (13.3%, *n* = 2), Asian (6.7%, *n* = 1), Non-Hispanic Black/African descent (6.7%, *n* = 1), and mixed race/ethnicity (6.7%, *n* = 1). Notably, all participants in the qualitative analyses also met criteria for inclusion in the high risk for ADHD group based on the quantitative analyses in phase one of the project, which is described in greater detail below. Table 1 summarises the descriptives for participant demographics included in the quantitative and qualitative analyses.

**Table 1.** Quantitative Participant Demographics (*n* = 307).

Demographic variables	*n*	%
Racial/ethnic background		
Non-Hispanic White or European	179	58.5
Arab or Middle Eastern	33	10.7
Asian or Asian descent (non-Arab)	32	10.5
South Asian or Southeast Asian	24	7.8
Black/African descent/Caribbean	16	5.2
Hispanic/Latino	4	1.3
Aboriginal/Indigenous/Metis	4	1.3
Other/mixed	15	4.7
Employment status		
Unemployed	83	27.0
Employed part-time	176	57.3
Employed full-time	48	15.6
Relationship status		
Single	139	45.3
In a romantic relationship	151	49.2
It’s complicated	17	5.5
Highest level of education		
High school diploma or equivalent	202	66.0
Greater than secondary school	105	34.0
Living situation		
Living alone	26	8.5
Living with family or romantic partner	249	81.1
Living with friends/roommates	32	10.5

### Quantitative Phase Procedures

All participants completed an online survey via Qualtrics, including the collection of demographic information. Community sampling involved email recruitment through the Canadian ADHD Resource Alliance (CADDRA) and The Centre for ADHD Awareness Canada (CADDAC) website, social media (e.g., private Facebook groups), and ADHD online support groups. Survey links were sent via email.

Upon clicking the survey link, participants were directed to the screening questionnaire. Those who met the inclusion criteria were automatically directed to the consent form, which detailed the study procedures and the incentives for participation. Respondents could only participate one time. Research pool members received extra credit in courses in which they were enrolled, and community participants were eligible for a draw for a $50 gift card. After their participation, all respondents received information about mental health resources.

### Quantitative Phase Measures

#### Adult ADHD Self-Report Scale (ASRS)

The ASRS is an ADHD screening tool appropriate for use in the general population (Kessler et al., 2005) and features 18-items with responses on a 5-point Likert scale ranging from 0 (“never”) to 4 (“very often”). The items closely map onto the diagnostic criteria for ADHD. The ASRS has demonstrated strong psychometric properties, including strong internal consistencies and moderate concurrent validity with other widely used ADHD rating scales (Adler et al., 2019). The ASRS scores were used to form the groups in the quantitative analyses in the first phase of the project and to describe those contacted for the second phase.

In addition to the ASRS, participants were asked if they ever received a formal diagnosis of ADHD on the demographics survey. If they responded affirmatively, they were asked about age of diagnosis and if they ever received treatment (never, only in the past, and in the present). Responses to this question were used to determine which participants to invite to participate in the second phase of the project.

#### Depression Anxiety Stress Scales-21 (DASS-21)

The DASS-21 (Lovibond & Lovibond, 1995) is a shortened version of the DASS-42, a non-clinical measure of distress associated with anxiety, depression, and stress symptoms. Respondents were asked to rate the accuracy of each statement over the last week. The 21 items feature responses on a four-point Likert scale, ranging from 0 (“Did not apply to me at all”) to 3 (“Applied to me very much, or most of the time”). Examples of statements include “*I felt I was close to panic*,” “*I found myself getting agitated*,” and “*I felt that I had nothing to look forward to*.” Higher scores reflect greater distress/symptoms. The DASS-21 is widely used, especially with university students and emerging adults. Psychometric support for the DASS-21 is also at least adequate, with strong support for internal consistency of the DASS-21 as well as good convergent validity with several clinical measures (Sinclair et al., 2012).

#### Difficulties in Emotion Regulation Scale-18 (DERS-18)

The DERS-18 (Hallion et al., 2018) is a short form for the original DERS 36-item scale (Gratz & Roemer, 2004). Items include statements such as “*When I’m upset, I become out of control*” and “*When I’m upset, I feel guilty for feeling that way*.” Higher scores represent greater emotional dysregulation. The DERS-18 demonstrated strong internal consistency (α = 0.92) and convergent validity with the full measure (Hallion et al., 2018).

#### Brief Resilience Scale (BRS)

The BRS (Smith et al., 2008) is a 6-item scale evaluating one’s ability to bounce back from stressful events. Respondents were asked to rate how much they agreed with statements such as “*I tend to take a long time to get over setbacks in my life*” and “*I tend to bounce back quickly after hard times.*” Responses were on a 5-point Likert scale ranging from 1 (“Strongly Disagree”) to 5 (“Strongly agree”). Higher scores represent greater resiliency. Internal consistency for the BRS has been demonstrated in a variety of populations and good criterion validity (Cosco et al., 2016).

#### UCLA Loneliness Scale—V3 (UCLA-LS)

The UCLA-LS (Russell, 1996) is a 20-item scale that measure a respondent’s feelings of loneliness and social isolation. Ratings range on a 4-point Likert scale from 1 (“Never”) to 4 (“Often”). Items include statements such as “*How often do you feel that there are people who really understand you?*” and “*How often do you feel alone?*” Higher scores reflect greater loneliness. It has been demonstrated to have strong psychometric properties, including high internal reliability, high test-retest reliability over a year, and adequate convergent/construct validity (Russell, 1996).

#### Self-Compassion Scale (SCS)

The SCS (Neff, 2003) is a 26-item scale measuring self-compassion, a construct involving how respondents respond to themselves difficult times. Responses are on a 5-point Likert scale, with answers ranging from 1 “Almost never” to 5 “Almost always.” Examples of items include “*When times are really difficult, I tend to be tough on myself*” and “*I’m tolerant of my own flaws and inadequacies.*” Higher scores reflect greater self-compassion. The scale has been demonstrated to be a reliable and valid measure of self-compassion (Neff, 2016).

### Qualitative Phase Procedures

All phase one participants were asked if they were willing to be contacted to participate in future research. Those who reported having an ADHD diagnosis and were willing to be contacted were invited to participate in the follow-up qualitative study. Completion of the second survey qualified them for entry into a draw for another $50 gift card. They were also provided with information about ADHD, including online resources.

The qualitative interviewing was also completed in Qualtrics via semi-structured written interview immediately after the informed consent process. In addition to offering greater flexibility in terms of geographic area of recruitment, online modalities may offer greater ecological validity in younger adults as they may be more comfortable disclosing information online compared to face-to-face (Shapka et al., 2016). Other studies have reported that the data collected from online interviews is comparable in quality to in-person methods with responses that were equally reflective and detailed in both conditions (Heerwegh & Loosveldt, 2008).

The interview focussed on the impact of the pandemic on participants and their coping behaviours during the pandemic. Qualitative analyses were used to answer the following questions: (1) What are the overlapping themes in relation to resilience, emotion regulation, and successful coping? and (2) What role do ADHD symptoms play in coping with very high levels of stress? To facilitate detailed responses, themes from the Brief-COPE (Carver, 1997) were used as prompts following open-ended questions about maladaptive and adaptive coping. The items make up 14 subscales representing distinct coping strategies (e.g., distraction, denial, venting, substance use, humour, religion, etc.). Items include statements such as “*I’ve been turning to work or other activities to take my mind off things*” and “*I’ve been using alcohol or other drugs to make myself feel better.*” Specific items or valuations, such as adaptive and maladaptive, from the Brief-COPE were not used. Instead, the coping themes were integrated into the semi-structured interview to prompt further elaboration. For example, participants were asked to click each strategy they used during the pandemic (e.g., “journal to express myself,” “use drugs or alcohol,” etc.). For each item endorsed, participants were asked to elaborate on the frequency and effectiveness of each strategy used in an open text box.

Content analysis, a method for identifying patterns and themes (White & Marsh, 2006), was used to illuminate common elements of coping during an unprecedented time. Responses were coded according to themes using well-established procedures (Erlingsson & Brysiewicz, 2017; Hsieh & Shannon, 2005). Specifically, responses were first reviewed to ascertain a general understanding of the central ideas or points made by participants, with pandemic-related coping as the phenomenon of interest. Every instance representative of a coping strategy was highlighted in the text and then divided into smaller, more meaningful units of data. Next, units of data were labelled with a code that reflected the core meaning of the unit. Codes that fit together were then grouped into categories, which were further interpreted into emergent themes. In other words, the underlying or latent meanings were extracted to create overarching themes. All coding was completed using a combination of Microsoft Word and Excel with data visualisations created using https://flourish.studio/ from Kiln Enterprises Ltd.

The unit of sampling was the full narrative within the survey, which was grouped by question (unit of data collection and analysis). The process of coding was iterative and inductive, with an initial coding scheme developed from the data itself. As the initial coding structure was not completely clear, existing theoretical frameworks were then utilised for guidance in a deductive fashion. The circumplex model of coping (Stanisławski, 2019) and the conceptual framework developed by Chun et al. (2006) were used to further develop coding categories and themes. This coding scheme was used to label all data and create a model of coping described by the participants.

#### Model of Coping

The model featured two bipolar dimensions of coping that intersect to create four quadrants. The first dimension (internal-external) reflected whether coping strategies are internally or externally focussed (Tweed et al., 2004). Internally focussed strategies included any behaviour that directly or indirectly addresses or avoids one’s internal experience, whether that be thoughts, emotions, or physiological sensations. This included efforts to modulate or escape emotional or physiological arousal by engaging in breathing exercises, cognitive reframing, denial, or suppression. In contrast, externally focussed strategies included efforts that are directed outside of the self. This included behavioural strategies such as actively addressing or avoiding the problem, redirecting attention away from internal experience (i.e., distraction), or seeking support from others. Although similar to the problem versus emotion-focussed model, the use of an internal-external dimension is more comprehensive as it encompasses a broader range of strategies.

The second dimension (approach-avoidance) defines whether coping efforts are directed towards or away from the perceived threat. Approach strategies include direct efforts to address or remove the stressor and mitigate distress (Carver & Connor-Smith, 2010). In contrast, avoidance strategies include behaviours (e.g., safety behaviours) or actions that avert direct engagement with the stressor, thereby reducing distress in the moment (Carver & Connor-Smith, 2010).

Each quadrant of the model represents the intersection of adjacent dimensions. Although similar to Chun et al.’s (2006) framework, it does not include their third dimension (collective vs. individual). The proposed model avoids the use of values or valence (positive/negative or adaptive/maladaptive) and instead, focusses on the broader context of participant responses and the words used.

Following from the model, a codebook outlining the structure of codes, categories, and themes was created. A secondary coder was trained using the codebook to establish inter-rater reliability. Numerical values were assigned to unique codes and Cohen’s Kappa indicated strong inter-rater reliability (*Κ* = .83, *p* < .001). There was 82% agreement (*n* = 113) for the 131 cases. Disagreement occurred when the raters assigned different codes to a statement or meaning unit. For example, the quote “*I've picked up cross stitching, which is my most cathartic hobby*.” was coded as *enjoyable activities/hobbies* by rater 1 and *learning/skill development* by rater 2. Any disagreement between raters was resolved through discussion until consensus agreement was achieved. Some codes were ambiguous and fit under multiple categories of coping strategies depending on the context (e.g., crying as emotional release vs. instrumental crying to elicit social support). In the case of ambiguous meaning units, items were coded based on the context and wording or how they were described. For example, to differentiate between socioemotional support and social diversion, the context was used to determine whether the goal of social engagement was for pleasure or for comfort. The following excerpts illustrate the difference: “*When I’m unable [to cope], I reach out to family and friends*” indicated the intention to seek support. In contrast, “*Asking a friend to hangout or FaceTime*” suggested social interaction to be an enjoyable activity, rather than a request for support.

## Results

### Quantitative Results

#### Grouping Procedure

In the demographics form, participants were asked if they had ever been diagnosed with ADHD and if they had received treatment. Among participants, 58 reported they had been diagnosed with ADHD and five were unsure (all unsure individuals endorsed receiving treatment for ADHD at some point in time, so it was assumed they received a diagnosis). Of those 63 participants, 10 had never received treatment, 13 received treatment in the past, and 40 were receiving treatment at the time of their participation.

Due to concerns about underdiagnosis of females (Attoe & Climie, 2023; Hinshaw et al., 2022) and limited access to publicly funded psychological assessments in Canada, self-reported inattention symptoms typically associated with ADHD were used to assign membership two groups: low risk for ADHD and elevated risk for ADHD. Using the Kessler scoring criteria for the ASRS (Kessler et al., 2005) the scores for the items related to inattention were summed and then assigned a probability of the individual meeting diagnostic criteria for ADHD. We specifically used inattention symptoms as these have been highlighted in the literature of representing how ADHD is usually manifested in adult/emerging adult women.

Using a conservative interpretation of the Kessler criteria (Kessler et al., 2005), any score below 14 is unlikely to indicate ADHD, scores ranging from 14 to 17 indicate a low probability of ADHD, and any score of 18 or greater is in the high probability range. For this study, individuals with a score of 18 or greater were assigned to the elevated risk for ADHD group (“elevated”; *n* = 73) and those below 14 were assigned to the low risk for ADHD group (“low”; *n* = 234). Those with ASRS scores between 13 and 17 (*n* = 85) were not included in the quantitative analyses or in the description of the sample.

#### MANOVA Analyses

Analyses were completed using SPSS 30. The first multivariate analysis of variance (MANOVA) was used to test whether individuals with elevated risk for ADHD would also be at elevated risk for loneliness, emotional dysregulation, and negative emotional states during the pandemic compared to participants at low risk for ADHD. The second multivariate analysis of variance was used to test the hypothesis that individuals at elevated risk for ADHD would report lower self-compassion and resiliency compared to participants who were at low risk for ADHD.

Prior to planned data analyses, the data were checked for missingness and whether MANOVA were met. Analysis indicated that values were missing at random (Little’s MCAR test: χ² = 2,944.36, *df* = 2,891, *p* = .521). Although the variables were non-normal in distribution, the violations were minor (Pituch & Stevens, 2015). Data were analysed with and without outliers (*n* = 6) with no change in outcomes, so all data were included in the MANOVA analyses. Although multivariate normality was violated χ^2^ (6) = 78.20, *p* < .001, visual inspection of matrix scatterplots indicate data from each set of dependent variables were roughly elliptical. Independent of observations was assumed, as participants completed surveys only one time.

Results from the first MANOVA indicated an overall difference in psychological difficulties between the elevated risk and low risk for ADHD groups, Wilks’ Λ*F* = 30.64, *p* < .001, partial η² = .234. These findings represent a large effect size, indicating that more than 20% of the variance in the dependent variables was associated with ADHD symptoms. Follow-up analyses of variance indicated a significant impact of ADHD symptoms on measures of emotional dysregulation, negative affect, and loneliness with those in the elevated risk group reporting more difficulties in each domain.

Results for the second MANOVA indicated an overall difference in psychological resiliency and self-compassion between ADHD and non-ADHD groups, Wilks’ Λ*F* = 25.44, *p* < .001, partial η² = .141. These findings indicate a medium to large effect size. Follow-up analyses of variance indicated significant effects of ADHD symptom grouping on measures of resiliency and self-compassion with those in elevated risk for ADHD group reporting less resiliency and self-compassion. Follow-up analyses for both MANOVAs are summarised in Table 2.

**Table 2.** Group Differences From Follow-up Analyses.

Measures	Low risk mean (*SD*) (*n* = 234)	Elevated risk mean (*SD*) (*n* = 73)	*F*	η²
DERS-18	42.61 (19.97)	58.00 (12.59)	80.59*	.21
UCLA-LS	45.58 (9.97)	52.26 (8.07)	31.83*	.10
DASS total	36.91 (12.71)	51.26 (13.85)	67.78*	.18
SCS	74.92 (17.59)	61.34 (14.48)	43.08*	.12
BRS	19.04 (4.76)	14.81 (4.97)	35.84*	.11

*Note*. DERS-18 = Difficulty in Emotion Regulation Scale; UCLA-LS = UCLA Loneliness Scale; DASS Total = Distress Anxiety Stress Scale Total Score; SCS = Self-Compassion Scale; BRS = Brief Resilience Scale.

* *p* <.001.

### Qualitative Analyses

#### Internal-Approach Strategies

Internal-approach strategies are direct efforts to engage with or address an internal experience, including emotions, physical sensations, or thoughts. Participants described using various cognitive strategies, such as cognitive reframing to manage distressing thoughts about the pandemic, which reflect internal-approach strategies. With this pattern, participants changed their interpretation of the situation to perceive it as more manageable. For example, some people viewed the pandemic as a reprieve from the demands of normal life: “*Beginning of the pandemic (e.g., March 2020), I was beginning to feel run-down from demands from school so having to completely stop for a few weeks was a nice unexpected break. Having time-off where I was forced to stay at home and not have any work to do was hard at first but made me realize I was taking on too much*.” (P8). Acceptance indicated that the participant reported making space for feelings of discomfort. For example, one participant said, “*Not try to run from my bad mood, if I was sad then I was sad and it’s okay to sit with that.*” (P13). Several participants reported expressing their emotions, for example “*I started going to therapy and writing down my feelings in a notebook.*” (P1). In this context, the implication is that the participant has begun sharing their emotions (either in writing or by talking with a therapist). A few participants expressed confidence in their ability to self-soothe and manage adversity: “*I have. . . . decent coping skills. After years of therapy I have become relatively self-sufficient and am able to guide myself through difficult situations.*” (P13). Some internal-approach strategies involved physiological regulation efforts—activities that can downregulate stress hormones and upregulate dopamine and endorphins, such as exercise, relaxation techniques, and meditation. For instance, one participant said, “*I took walks to ease the feelings of stir craziness and tried to do exercise videos.*” (P12). Another noted, “*I work out, go for a lot of walks.*” *(P3).*

#### External-Approach Strategies

External-approach strategies are attempts to address a problem or emotional discomfort through means outside of the self. These strategies included engaging in active problem-solving. Several participants noted that routines and social interaction were helpful. For example, one person said: “*Work/job permitted me to maintain a routine schedule where I still had to leave the house to do my job instead of working from home. Allowed me to maintain interactions with my colleagues and be able to be around people who were forced to endure the same struggles working on the job during the pandemic (as people who work at a pharmacy that located inside one of the major grocery retailers). This created a stronger group cohesion and have a great support system at work. As someone who actually enjoys working at her pharmacy, I think this helped me cope better and forced me to maintain proper sanitary (e.g., shower regularly) and sleep routines (e.g., had to sleep at a decent hour in order to be prepared for work).*” (P8). Thus, this individual experienced substantial benefits from the opportunity to be at work in an environment with colleagues.

Not all participants had the opportunity to work. Some described engaging in household chores: “*CLEAN. I cannot understate* [sic] *how much of a mood booster/how satisfying it is to finally get to cleaning the bathroom you’ve been avoiding*” (P13). Participants also reported talking to family and friends or seeking counselling: “*I call help lines, specifically kids help phone a lot because it feels safer than other ones. I go to counselling. I talk to friends online more*.” (P14). This was categorised as socio-emotional support, which refers to seeking out others for comfort, meaningful connection, guidance, or access to resources such as financial support. Although seeking emotional support may involve efforts to stave off distressing internal experiences, such as feelings of loneliness and isolation, the individual sought comfort outside of themselves. While the outcome of therapy may be internal strategies (e.g., cognitive reappraisal, acceptance, or emotional expression), in this case, it is categorised as an external strategy because the individual seeks out support or guidance to achieve such outcomes.

#### External-Avoidance Strategies

External-avoidance strategies involve shifting attention away from the problem for temporary distraction from stressors or distress. In other words, attention is shifted outward towards some external stimulus to produce relief. Strategies reported by participants included social diversion, engaging in enjoyable hobbies or activities, and distracting oneself with different forms of entertainment, like reading or watching movies. Social diversion referred to engaging in social interaction for recreational purposes, as opposed to seeking support or comfort. For example, one participant said, “*Asking a friend to hangout or FaceTime (especially while working from home).*” (P13). Many participants reported engaging in various hobbies and entertainment activities: “I’ve picked up cross stitching, which is my most cathartic hobby.” (P3); “*I distracted myself with social media, youtube, netflix, video games, etc.*” (P2). Some participants preferred to focus on school or work: “*working hard and keeping busy have all contributed to my managing well during the pandemic.*” (P3). Overall, external avoidance strategies entail distraction or escape from the distressing experience.

#### Internal-Avoidance Strategies

Internal-avoidance approaches include efforts to modulate or circumvent some internal experience, for example distressing emotions or physical tension in favour of more pleasant ones. Several participants reported avoiding pandemic-related news or content: “*It was also helpful to stop watching and obsessing over the news, get off social media, and focus on things other than the pandemic*” (P12); “*Consuming positive media or media produced well before the pandemic. Allowed me to distance myself from the pandemic and not get caught up in ruminating on my lack of power.*” (P4). Effort to improve subjective well-being by avoiding distressing information about the pandemic was categorised as hedonic disengagement.

Participants described other ways in which they turned away from distress and towards more pleasurable experiences. For example, a few participants described using substances when other efforts failed: “*My use of cannabis increased significantly; both in frequency and quantity. I found reaching out to friends to be tiring sometimes due to ‘zoom fatigue’ and disliked how much screen time I was participating in.*” (P2). In this case, it appears that cannabis use increased because the usual coping methods (i.e., socioemotional support) contributed to feelings of burnout. Some participants reported seeking comfort through food: “*Increase junk food intake (instant relief but did not feel good after).*” (P8). Others reported engaging in activities that centre around relaxing and calming distraction, such as “*calming herbal teas. . .listen[ing] to music*” (P13). Attempts to escape distressing feelings by pursuing activities eliciting temporary moments of pleasure were categorised as hedonic escapism. For example, participant 12 said, “*I also made lots of online purchases.*” Although hedonic disengagement and hedonic escape are similar concepts, they appear to represent varying degrees of withdrawal (nonengagement vs. complete escape).

### Use of Strategies

Overall, participants were most likely to use external-avoidance strategies the most, followed by internal-approach, external approach, and internal-avoidance. Table 3 summarises these results. Even though external avoidance strategies were the most frequently endorsed, respondents rated internal-approach strategies to be the most effective overall. External-avoidance strategies, such as distraction, were rated as the second most effective, but more so in the short term. In other words, distracting oneself from problems/stressors only provided momentary relief—as opposed to dealing with the root of the problem. Overall, respondents were less likely to use and report benefits from internal-avoidance approaches.

**Table 3.** Joint Display of Participant Coping Strategies Use and Effectiveness.

Strategy theme	*k*	*M* (*SD*)	Effect	Exemplar quote
External avoidance	59	3.93 (1.62)	80	“I took up new hobbies that I would be able to do at home. I did lots of zoom meetings with family/friends.” (P12)
Internal approach	46	3.06 (2.02)	93	“Not try to run from my bad mood, if I was sad then I was sad and it’s okay to sit with that.” (P13)
External approach	35	2.33 (1.50)	76	“I call help lines, specifically kids help phone a lot because it feels safer than other ones. I go to counselling. I talk to friends online more.” (P14)
Internal avoidance	27	1.80 (1.01)	44	“I still struggle with instinctively wanting to go to food to deal with discomfort which I know is not healthy.” (P11)

*Note. N* = 15; *k* = prevalence of reported strategies; Effect = the total rating of effectiveness with higher scores representing greater effectiveness. Prevalence of strategies reported by participants to cope with the pandemic.

## Discussion

The goal for this mixed methods study was to describe how women with ADHD coped with pandemic stress. As anticipated by the hypotheses for the quantitative phase of the study, people with more symptoms commonly associated with ADHD were lonelier, more emotionally dysregulated, and experienced more negative emotional states compared with people who were at low risk for ADHD. The results also indicated that having more ADHD symptoms was associated with less resilience and self-compassion.

These quantitative results corroborate previous findings regarding the impact of the pandemic on emerging adults’ mental health (Dragioti et al., 2022; Lu et al., 2022). Further, the results also support concerns that individuals with more ADHD symptoms represent a vulnerable population compared to those with few or no symptoms, particularly during a period of high stress. These results are in line with other studies (Bailie & Linden, 2024). Emerging adult women with ADHD symptoms may face unique challenges during times of heightened turmoil, such as a global health crisis. What is not necessarily documented in the literature is the coping strategies used by women with ADHD when they are under significant stress.

### Coping and Resilience

Following from the quantitative results, participants who indicated past diagnoses of ADHD were interviewed about their coping and resilience. Participants who reported managing well during the most restrictive phases of the pandemic attributed this to the use of strategies that engaged with the stressor or distress, namely approach strategies. Internal-approach strategies reported by participants included cognitive reappraisal, acceptance, emotional expression, and physiological regulation. The latter included any activity that downregulated stress hormones and upregulated dopamine and endorphins. These activities include exercise, relaxation techniques, and meditation.

Whether individuals are consciously aware of it or not, physical activity improves mood and subjective well-being, as it indirectly regulates physiological arousal (Mikkelsen et al., 2017). Similar effects have been observed following mindfulness meditation and relaxation training. Previous research has indicated that biomarkers of stress, including elevated inflammatory cytokines and high blood pressure treatment, are reduced with focussed meditation or relaxation training (Hoge et al., 2018; Yilmaz & Bulut, 2020). Thus, some internal approach strategies likely impacted biological stress processes.

Our results also indicate that those who found internal-approach strategies to be more effective reported higher ratings of self-kindness and resilience, and lower ratings of depression symptoms and emotional dysregulation. Our results extend previous research connecting mental health and resiliency (Liu et al., 2020), with internal-approach strategies that engage with or move through distressing situations and/or emotions also are associated with greater self-compassion and increased capacity to regulate emotional arousal. These internal approach strategies were also associated with resilience, a state defined by the ability to respond flexibly to challenges and bounce back from adversity (Lazarus & Folkman, 1984). Thus, not only is emotion regulation tied to resilience, but the findings also suggest that emotion regulation strategies that specifically engage with one’s internal experience are linked to successful coping during a highly stressful period in the recent past for women with ADHD.

It can be argued that the overarching pattern within approach strategies relates to the individual’s perceived ability to manage or tolerate distress without exceeding their ability to cope. This is consistent with Lazarus and Folkman’s (1984) cognitive model of threat appraisal and coping. They posited that when coping abilities are perceived to be greater than the demands, the stressful situation is considered a challenge to be overcome. In contrast, situations in which demands outweigh available coping resources may be identified as a threat—evoking feelings of fear, anxiety, or even anger. This may suggest a connection between self-efficacy and the tendency to use approach strategies, as the stressor is perceived as a challenge to be directly addressed.

### Depths of Experiencing

It may also be helpful to consider how deeper levels of experiencing predict resiliency during difficult times (Elliott et al., 2014). Instead of analysing how participants described their difficulties and coping strategies, a depth of processing framework can be applied to the coping themes directly. Specifically, it can be argued that each quadrant (or theme) of the proposed model reflects various levels of emotional processing. For instance, avoidance strategies are like lower levels of experiencing when emotional experiences are kept at a distance. Avoidance strategies move away from the distressing emotion or situation and effectively hold the person in emotional stasis. Rather, attention is shifted away from the stressor, while the upsetting emotions or thoughts are unchanged. Participants who reported external-avoidance strategies, such as distraction, to be effective during acute moments of distress also noted that relief was often temporary. Distress reportedly returned as soon as respondents stopped engaging in distraction.

Internal-avoidance strategies go one step further in that they aim to increase pleasant experiences in addition to avoiding unpleasant ones. For instance, engaging in cannabis consumption can dampen the intensity of unpleasant emotions like anxiety and physiological sensations like hyperarousal, while inducing feelings of relaxation and euphoria (Vorspan et al., 2015). Following the depths of processing framework, it might be argued that internal-avoidance strategies reflect poor engagement with the underlying emotional experience, in line with hedonic escapism, which involves escaping from everyday life through transient moments of pleasure (Holmqvist et al., 2020). In the context of the pandemic, everyday life may include feelings of uncertainty, persistent anxiety, and loneliness. Thus, hedonic escape involves activities that provide momentary relief from the discomfort of pandemic-related distress. Our participants endorsed using escapist strategies such as humour, suppression via substance use, comfort through food (i.e., emotional eating), soothing distraction, and shopping.

In contrast, approach strategies appear to reflect deeper levels of processing, whereby participants engage with or move through distressing emotions or problems. The current study’s findings are consistent with the depth of processing research: deeper levels of engagement with emotional processes are associated with better mental health outcomes, such as reduced depression symptoms. Yet, this should not be interpreted through a lens that suggests hedonic escapism is inherently maladaptive. It has been argued that disengagement can be adaptive when cognitive distancing is done in a flexible, intentional manner in conjunction with the ability to maintain interpersonal relationships (Denckla & Bornstein, 2015) and when avoidance of problem-related information allows an individual to bolster their propensity to sustain well-being over time (Stanisławski, 2019).

In the current study, hedonic disengagement also manifested as attempts to avoid excessive consumption of troubling news, which may be highly adaptive. Limiting news consumption during the pandemic was associated with reductions in anxiety, stress, and feelings of despair (Mannell & Meese, 2022). This is consistent with participants’ reports, which included behaviours like going for walks, acquiring new hobbies, and avoiding the repetitive news cycle. Critically, there must be some balance between staying informed during a rapidly evolving crisis and restricting overconsumption of news (Mannell & Meese, 2022). Taken together, the results of our study suggest that internal avoidance strategies were helpful under specific circumstances, but overall, were the least effective for managing difficulties during the pandemic.

### Role of ADHD Symptoms in Coping

In general, participants with ADHD were more likely to use external-avoidance approaches, like distraction, despite reporting them to have a time-limited benefit. Participants acknowledged that internal-approach strategies were the most effective, yet they used external avoidance strategies more frequently. One possibility is that participants were experiencing acute distress, in which case, distraction was likely effective in the moment. Notably, distraction may be helpful in circumstances where emotional distress becomes overwhelming and there are no remedies available to the individual (Linehan & Wilks, 2015).

Another plausible explanation may be related to the connection between ADHD and emotional regulation difficulties (Kooij et al., 2019). Respondents who were less accepting of their emotions were more likely to use external avoidance strategies, while respondents with reduced emotional awareness were less likely to use internal approach strategies. It is possible that emotional dysregulation associated with ADHD may contribute to difficulties with confronting and tolerating distressing emotions, thoughts, and physical sensations, which may signal sympathetic nervous system arousal. There is also evidence that individuals with ADHD may be prone to impairments in self-awareness (Butzbach et al., 2021). Taken together, these results suggest that reduced self-awareness in combination with limited distress tolerance may hinder the use of internal approach coping strategies as external avoidance strategies may be a less daunting option, especially during times of acute distress.

The current study’s findings provide support for the use of internal-approach coping strategies—that is, strategies that engage with and modulate internal experiences. It may be that actively practicing making space for uncomfortable emotions increases the ability to respond more flexibly to challenges over time (Greenberg & Goldman, 2018). For women with ADHD who are already at heightened risk for mood and anxiety disorders (Fayyad et al., 2016), greater emotional awareness and practice with distress tolerance may become central to self-soothing and self-compassionate behaviours.

### Limitations

Although informative, there are several limitations that should be considered in the application of this study. Using online data collection presented several challenges. Substantial efforts were taken to minimise invalid responding (e.g., using the reCAPTCHA test, location validation, and specific features associated with Qualtrics), but there were remaining issues. For example, at one point the survey was inundated with spam bots and there were multiple responses from the same person and there were ineligible individuals completing the study. Ultimately, the data required careful screening and additional participants were recruited to protect the integrity of the study design. Because our design was also within the pandemic, generalisability to other scenarios may be limited to circumstances such as a global pandemic or to populations like our target sample, emerging adult women in North America with ADHD.

The decision to use symptom frequency to determine group membership instead of formal diagnosis for the quantitative analyses may be considered controversial. There are pros and cons to each approach. Using formal diagnosis as a criterion would have been a more conservative approach. The sensitivity of the study could theoretically be contaminated with women who did not actually have ADHD if symptoms alone were used as the criterion; however, relying solely on formal diagnosis might not fully capture all participants with ADHD in our sample. It is thought that many women go undiagnosed because their symptoms are not overtly disruptive, if they have limited access to care, or their coping skills exceed their difficulties, or they have learned how to cope with their symptoms (Attoe & Climie, 2023). Individuals with less obvious symptoms may not meet the threshold for diagnosis, despite experiencing impairment related to ADHD symptoms (Platania et al., 2025). On the other hand, symptoms like inattention and emotion dysregulation are not exclusive to ADHD (Katzman et al., 2017). Thus, scores above the ASRS cutoff do not necessarily mean they meet the criteria for diagnosis. To note, all data in this study emanate from participant self-report. Thus, shared method variance may have influenced the results. In sum, the design of the study may mean that the results are not necessarily representative of all women with ADHD.

### Implications

Since our data collection, COVID-19 has become endemic and pandemic restrictions have ended. Yet, public health officials have suggested that there will be more pandemics in the future (Casadevall, 2024). In addition, recent geopolitical events and growing world tensions are likely to cause substantial distress. Results from this study are consistent with other research that suggests those who actively engage with and modulate their experiences of distressing internal experiences, including emotions, thoughts, and physiological discomfort have better mental health outcomes and greater resiliency (Elliott et al., 2014). Unfortunately, individuals with ADHD may struggle to engage in these coping strategies due to deficits in emotion regulation and self-awareness. These findings provide clear targets for intervention directed towards women with ADHD.

There are many models of programming for people with ADHD being offered in-person and virtually, and in individual and group formats. These programmes have the potential to promote self-compassion and reduce stigma surrounding ADHD and emotional distress. Programme content may include teaching strategies to increase emotional awareness, distress tolerance, and self-soothing. Modules from dialectical behaviour therapy may also be used to help participants develop better emotion regulation and distress tolerance skills (Linehan & Wilks, 2015). Mindfulness-based interventions may offer another means to achieve these outcomes, as they may help individuals with ADHD develop important regulatory skills, including being attentive, non-judgemental, and employing relaxation strategies to reduce agitation (Nilsson, 2021). Interventions from acceptance and commitment therapy (Harris, 2019) may also be helpful for developing awareness of thoughts and emotions and cultivating emotional acceptance for women with ADHD coping with highly stressful situations.

The results from this study represent a contextually driven snapshot of the lives of women with ADHD during a global pandemic. Given the limited availability of vaccines at the time of the study, this was a time of acute distress for many individuals. Pandemic-related restrictions and lockdowns resulted in prolonged isolation, which is known to be deleterious on mental health and well-being. In addition to the implications for preparations for future crises predicted by public health experts, the post-COVID “normal life” is also substantially different than life prior to 2019. Studying the response to pandemic-related distress in vulnerable populations, such as individuals with ADHD, has implications for public health policy, including but not limited to preparedness for future pandemics. Thus, it is crucial that resources are in place to improve pandemic-response planning, including access to and provision of mental health services. It may be that enhanced access to mental health services may reduce the overall cost of future pandemics for all people as well as in the economic impact.
